# Using Eye Movements to Understand Sense of Control in Situated Action

**DOI:** 10.1111/cogs.70154

**Published:** 2025-12-28

**Authors:** Nils Wendel Heinrich, Annika Österdiekhoff, Stefan Kopp, Nele Russwinkel

**Affiliations:** ^1^ Institute of Information Systems (IFIS), Department of Computer Science and Engineering Universität zu Lübeck; ^2^ Cognitive Psychology and Ergonomics Technische Universität Berlin; ^3^ Social Cognitive Systems, Faculty of Technology, CITEC Bielefeld University

**Keywords:** Sense of Control, Situated action control, Eye‐movement control, Dynamic environments, Statistical modeling, Embodied cognition

## Abstract

This series of studies investigated the interplay between the Sense of Control, continuous action control, and eye‐movement behavior in dynamic and uncertain environments. Across three experiments, we used a custom‐designed environment combined with eye‐tracking to examine how action goal pursuit and visual strategies were adapted to deal with motor perturbations of varying predictability. Participants steered a spaceship, avoiding walls and obstacles while contending with random input noise and predictable horizontal drift. We found that changes in fixation distances to a reference point, the spaceship, indicated the type of action control employed. Input noise was associated with decreasing distances in fixations already close to the spaceship, addressing immediate demands for maintaining the spaceship's trajectory. In contrast, fixations allocated within the outer vicinity of the spaceship featured even longer distances in response to drift, suggesting visual exploration and proactive planning. That is, reactive strategies of action control were characterized by immediate responses to unpredictable disturbances, whereas proactive strategies reflected anticipatory adjustments to predictable changes. Furthermore, judgments about the own Sense of Control were closely tied to participants' ability to anticipate and adapt to environmental features. Invisible perturbations led to control loss and reduced task performance, but predictable perturbations allowed participants to maintain a high Sense of Control and still successfully solve the task. These results highlight how cognitive processes and sensorimotor control interact to navigate uncertain environments by flexibly balancing reactive and proactive strategies of action control.

## 1 Introduction

Actions are executed with the intention of achieving a specific outcome, the action goal. At any given moment, an agent is aware of the various outcomes it can achieve with actions. With *action outcomes*, we refer to the changes in the environment the agent can bring about. All these possible actions constitute the agents action field (Kahl, Wiese, Russwinkel, & Kopp, 2022). The cognitive process of choosing among these possibilities within the action field is known as action selection. However, the action field can vary significantly even under similar conditions, partly because actions rarely occur in isolation.

Human agents act within dynamic environments, performing sequences of consecutive actions. Thus, individual actions cannot be considered completely separate from one another. Naturally, the selection of each new action likely depends on how effective preceding actions have been. The evaluation of actions is based on the degree to which they achieved the intended action goal and whether difficulties arose during execution. Said difficulties relate to the violation of the predicted sensory consequences. While we perform an action, a forward model generates predictions regarding the sensory feedback associated with each of the individual motor commands that constitute the action. The prediction is compared with the actual sensory feedback. Mismatches indicate prediction errors, signaling a deviation from the planned course of action. But even if many prediction errors were encountered, an action can still achieve its intended action goal. In fact, agents can use the feedback from prediction errors to adjust and correct actions in real time (Synofzik, Vosgerau, & Newen, 2008). This is similar to cycling in strong side winds that can unexpectedly push the handlebars causing us to deviate from our intended paths. But one can easily correct by steering straight again and mostly stay on course. In human agents, leveraging feedback is discussed to be based on a phenomenal experience, the Sense of Control (SoC; Jeannerod, 2003; Pacherie, 2008). It entails both the feeling of being in control of an action and the need to exert control to maintain the course of an action when encountering difficulties (Pacherie, 2008). Composing action fields, action goal selection, predicting sensory consequences, and correcting the course of actions are all components of action control, and experiencing control loss could lead to an adaptation of any of these components. There are theoretical frameworks attempting to explain the link between the SoC and adaptation of action control, but testing these models remains a challenge, especially with regard to action control within dynamic environments.

To address this challenge, we designed a dynamic experimental environment and introduced conditions known to influence participants' SoC (Österdiekhoff, Heinrich, Russwinkel, & Kopp, 2024). We conducted several experiments using our custom dynamic environment in combination with eye‐tracking. We argue that it is possible to infer statistics of action goal properties from gaze patterns on the screen (Heinrich, Österdiekhoff, Kopp, & Russwinkel, 2024). Measuring changes in fixational eye movements in response to the different experimental conditions thus allows us to investigate how the SoC and action control are connected.

### 1.1 Sense of Control

The SoC is a crucial component of the broader Sense of Agency, which also includes a sense of being the cause of an action and a sense of having initiated the action in the first place (Pacherie, 2008). The Sense of Agency in its entirety would lead to an agent assuming ownership for the action, which enables differentiating between self‐generated and externally caused events which fundamentally links to our perception of self.

In literature, it is mostly assumed that the SoC is based on the comparison between predicted sensory consequences of a motor command and the actual sensory feedback (Synofzik et al., 2008). If prediction and feedback match, the SoC is increased (in turn leading to a high Sense of Agency, as discussed by Blakemore, Wolpert, & Frith, 2002; Haggard, 2005; Tsakiris, Prabhu, & Haggard, 2006); conversely, it diminishes when prediction errors in sensory states occur.

But the comparison can go beyond mere sensory states. It can also assess the match between the intended action goal and the actual environmental state that was brought about by the action. This comparison is on higher conceptual levels than the one identifying prediction errors, as it requires the agent to interpret the state it is in. Comparing intended and achieved outcome refers to the *consistency* criterion of the postdictive account of agency (Wegner, 2003; Wegner & Wheatley, 1999), which assesses whether an action has successfully achieved its intended goal. The conclusion can, of course, only be drawn once the action is completed. This aspect of goal completion is likely what individuals recall when asked to retrospectively judge their own control (Chambon & Haggard, 2012). If the consistency criterion is not met, lower values for control judgments are expected. The distinction between prediction errors and the consistency criterion is crucial, because while both affect the SoC, it is quite possible that they trigger different types of action control adaptation.

### 1.2 Action control

Frameworks explaining action control often draw heavily from motor control accounts and almost all of them assume a forward model that predicts the outcome of the planned motor program, and additionally, an inverse model which infers the sequence of motor commands that will achieve a desired state (Cooper, 2010). This theory of model‐based motor control, therefore, relies on mental representations of the environment to link motor commands to sensory states and desired outcomes. But it also explains mechanisms such as error detection, feedback processing, and sensory integration that underpin short‐term motor adaptation and longer‐term motor learning (Jordan, 1996). The feedback at the core of these mechanisms can be extrinsic (e.g., explicit guidance or information on performance), but in this work, we will focus on intrinsic feedback (specifically through proprioception and vision).

One more recent and comprehensive framework that links intrinsic feedback and adaptation in action control is proposed by Kahl et al. (2022). The authors describe action control as adapting through recurrent feedback loops in situations of reduced control. They refer exactly to the comparator mechanism of which the output is fed into the SoC of the agent and link it to action selection in particular. Kahl et al. distinguish between two types of SoC, each corresponding to a different level in the action control hierarchy. The comparison between sensory states is associated with a low‐level SoC that is located at the sensorimotor control layer. In contrast, the comparison between intended and actual action outcome is linked to a high‐level SoC that in turn is associated with higher cognitive control levels. If either comparison identifies a mismatch, the respective SoC is decreased. Additionally, a considerable drop in low‐level SoC also causes a decline in high‐level SoC. The authors discuss how this dynamic high‐level SoC influences top‐down (goal‐directed) action control by restricting the composition of the action field in situations of control loss. Meaning that in challenging situations, the action field will only consist of action possibilities that are more likely to be accomplished given the constraints of diminished SoC (i.e., less likely to elicit prediction errors or fail to meet the consistency criterion). Because the action goal is selected from the action field, consequently, the selected action goal is bound to the SoC in the given moment. However, Kahl et al. did not specify what exactly this means for the properties of action possibilities that more likely constitute the action field and the selected action goal. Specifically, regarding the action goal properties, we intend to provide experimental results and, in addition, explore a new aspect of anticipation that has not yet been discussed.

Finally, Kahl et al. do assume that the sensorimotor control layer is highly capable of responding to drops in low‐level SoC all on its own. As in the example of cycling in strong winds, small adaptations in motor control can already overcome the difficulties. With the experiments described in this work, we cannot reliably distinguish between action control adaptation that is elicited by lower or higher levels of the action control hierarchy. Therefore, we will not attempt to map the different ways of adapting on specific levels, but we will discuss different types of action control adaptation.

The mechanisms described above only cover one type of action control adaptation: *reactive* adaptation of action control as it is action control that is adapted in response to control loss (Meiran, Cole, & Braver, 2012). But an agent can already anticipate a challenging situation with an associated decline in SoC and prepare itself accordingly for the upcoming circumstances. Here, the agent engages in *proactive* adaptation of action control, yet this presumes that it has a high degree of control. An increased SoC means that the forward model is accurate that in turn generates accurate predictions. Thus, proactive adaptation of action control is based on the ability to correctly project the circumstances of an upcoming situation. The agent then controls its actions in a way that is directed toward goal completion even under more challenging conditions. This illustrates the bidirectionality of the system. The SoC arises from action control, but also feeds back into action control. Despite extensive research on the phenomenological experience of control, few studies have explored how this experience impacts action control. In this paper, we address the research question of how action control changes with different degrees of SoC. Nevertheless, this poses the challenge of how to measure shifts in action control.

### 1.3 Inferring action goals from eye movements

In dynamic environments, it is almost impossible to predict when agents will select an action goal and start pursuing it, as individual instances are not explicitly separated from each other. Likewise, action goals can be abandoned at any time to initiate a new action selection process. To better understand human behavior in these settings, we need a reliable way to measure action goals. Visuomotor control theories suggest that visual focus can effectively guide motor responses, resulting in a seamless integration of visual perception and motor actions (Hayhoe, 2017). This can be observed, for example, in many simple mundane activities (e.g., making tea; Land, Mennie, & Rusted, 1999), but is also heavily researched in natural driving, which features a much more complex and dynamic environment. Research shows that drivers tend to steer toward the point they are looking at (Cina & Rad, 2024; Wilkie & Wann, 2002). Steering is visually guided by focusing on journey points on the road ahead, and as the car reaches these journey points, the gaze shifts further ahead again (Land & Horwood, 1995). However, drivers do not keep their gaze fixed on journey points; they periodically sample from various parts of the road (Kandil, Rotter, & Lappe, 2009; Wilkie & Wann, 2002). Additionally, drivers' gaze is directed not straight at journey points but also at other targets important for the driving maneuver, such as other cars during overtaking or the inside of a curve while turning (Lappi, 2022; Land & Lee, 1994). The final gaze location, therefore, is a synthesis of the journey point and the other targets. Even though it may be difficult to directly infer action goals from gaze locations, the current action goal significantly influences where the gaze is directed (Heinrich et al., 2024). This means that tracking the eyes of participants in a navigation task, should enable us to statistically differentiate between action control adaptation that is reactive or proactive in nature. And if we can identify distinct types of action control adaptation, we can attempt to assign them to the different experimental conditions that we know have influence on the SoC.

For the purpose of this work, we have to make specific assumptions about the connection of reactive and proactive adaptation of action control and changes in eye‐movement behavior. In the context of continuous action control, reactive adaptation could mean that action goals are selected with shorter time horizons; they target intended outcomes that are not as far ahead in time, as predicting the environmental state becomes increasingly uncertain with more time steps. This would mean that the eyes would be allocated closer to a reference point that is the start of a planned course of action. Proactive action control, on the other hand, would imply that the eyes fixate more strongly on certain areas in which challenging conditions are anticipated. We specifically assess fixational eye movements of participants controlling an agent in a custom‐designed experimental environment. We investigate the extent to which the statistics of these fixations can be explained by unpredictable and predicable features of the environment while accounting for the complexity of the visual scene. The resulting differences should also align with self‐assessments of control (Judgment of Control; JoC).

## 2 Methods

### 2.1 Setup

Participants were seated comfortably in a chair with a front rest with their heads placed in a chin rest at a height of 23.2 cm on a height‐adjustable table. The task was presented using a 28'' ASUS PB277Q screen with a 60 Hz refresh rate and a resolution of 1920x1080 pixels. The monitor was placed on the table at 80 cm distance from the participants head, with the center of the screen slightly below eye level. A high‐frequency eye‐tracking camera was positioned below the screen in 26.4 cm distance to the eyes. For task input, a keyboard on which the relevant keys were marked was placed directly in front of the participants.

### 2.2 Dodge Asteroids environment

Inspired by the simulation environment of Kahl et al. (2022), we have designed a custom experimental environment called the *Dodge Asteroids* environment^1^ (Abalakin, Heinrich, Österdiekhoff, Kopp, & Russwinkel, 2024; Heinrich, Russwinkel, Österdiekhoff, & Kopp, 2023; Österdiekhoff, 2023). It is implemented in Python (Van Rossum & Drake, 2009) using the PyGame package (Shinners & Pygame Community, 2000) and runs at 60 FPS. A spaceship of 36 pixels in width and length automatically traverses downward in an environment of 720 pixels width and length of up to 18,000 pixels. The free fall of the spaceship is 6 pixels every time frame. Participants can press either Y or M on the keyboard to move the spaceship 6 pixels to the left or right, respectively. There are walls on both sides of the environment. Obstacles of 36 pixels in width and length are randomly scattered throughout with their x and y coordinates sampled from a uniform distribution bounded by width and height of the environment. The goal in each trial is to steer the spaceship across the finish line at the bottom of the environment without crashing into walls or incoming obstacles. The finish line is indicated by a green line ranging horizontally from one wall to the other.

The Dodge Asteroids environment features two experimental manipulations.

Input noise can make it more difficult to control the spaceship. If a key is pressed to move the spaceship horizontally, in each game frame the key is down, the step is sampled from a normal distribution centered over the usual step size of 6 pixels and with varying standard deviation. This means that with increasing standard deviation, there is more uncertainty linked to the horizontal movement of the spaceship. This will evoke prediction errors in individual sensory states in the short term, but in the long term will also lead to a failure of meeting the consistency criterion when pursuing action goals.
Drift is indicated by red bars of 18 pixels width outside the walls, 45 pixels away. Drift sections span 270 pixels in vertical direction exactly the length of the red bar. As soon as the spaceship enters the drift section, it is moved by 3 pixels (exactly half the step size when a key is pressed) in a specific horizontal direction in each game frame. The direction of the horizontal movement is indicated by the side on which the red bar is displayed: If the red bar is displayed on the left‐hand side outside the walls, the spaceship is pushed to the right and vice versa (comparable to wind, which emanates from the drift bar and acts on the spaceship). The positions of drift sections within the environment as well as drift directions are randomized. The point in time of the onset of drift, as well as its effect, is predictable. We use this manipulation to investigate the role of (missing) prediction errors while still affecting the steering of the spaceship.

**Fig. 1:**
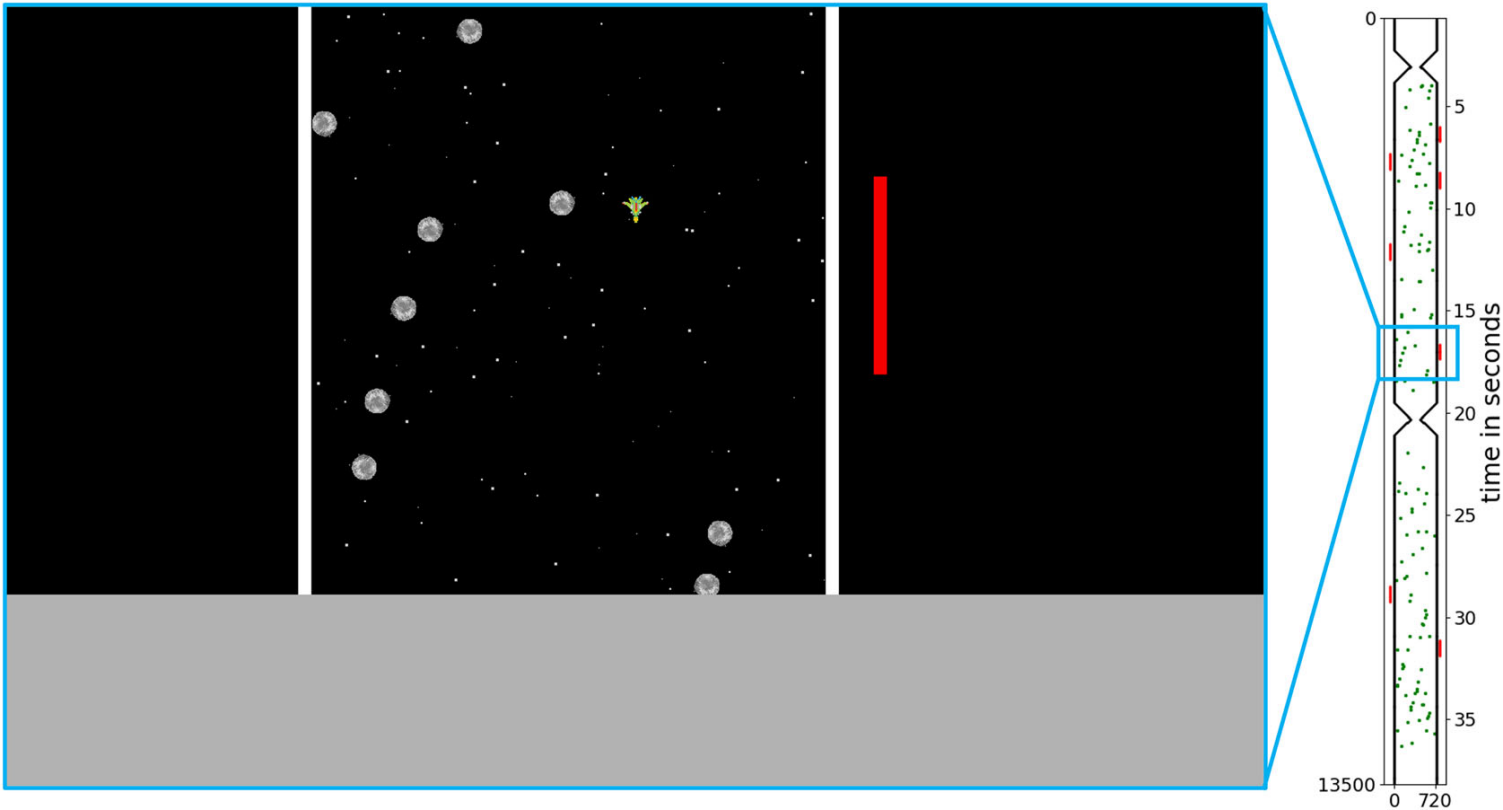
Instance within the Dodge Asteroids environment (based on the figure by Heinrich et al., 2023). The green spaceship, fixed at the horizontal center of the screen, serves as a reference point for fixational eye movements. Obstacles are spread throughout the instance. The red bar indicates a drift section, in which the spaceship is pushed to the left. The full layout of this trial is shown on the right.

For Experiment 1, we randomly generated six different layouts of the Dodge Asteroids environment, defined by where obstacles and drift sections are located. Three of the layouts had a length of 9000 pixels (short), the other three have a length of 18,000 pixels (long). We introduced three different degrees of difficulty (easy vs. medium vs. hard) depending on the number of obstacles and drift sections. The number of obstacles in short layouts ranged from 12 (easy), over 34 (medium) to 68 (hard), whereas in long layouts, it ranged from 32 (easy), over 84 (medium) to 168 (hard). The amount of drift in short layouts ranged from four drift sections (easy & medium) to eight drift sections (hard), whereas in long layouts, it ranged from nine drift sections (easy & medium) to 18 drift sections (hard). An example layout is shown on the right‐hand side of Fig. 1. Each layout was played with each of the input noise settings while drift could be on or off resulting in a 3x2 design: input noise (with standard deviations of 0, 3, and 6 pixels referred to as none, weak, and strong input noise, respectively) x drift (on vs. off). Therefore, each layout was played in a total of six different configurations, resulting in a total of 36 different configurations.

For Experiment 2, we randomly generated six layouts of the same length, 13,500 pixels. Three of difficulty medium (58–62 obstacles), and the other three of difficulty hard (116–124 obstacles). We did not include drift in Experiment 2. In return, we did introduce two further levels for input noise. The standard deviations of these additional levels were 9 and 12 (0, 3, 6, 9, and 12 pixels of standard deviation). Each layout was played with each of the five different input noise levels, resulting in a total of 30 different configurations.

For the latest iteration of the Dodge Asteroids environment, Experiment 3, we again random generated six layouts of 13,500 pixels length. Here, also three layouts were of medium difficulty (58–62 obstacles) and the other three were of hard difficulty (116–124 obstacles). Every layout regardless of difficulty contained eight drift sections at fixed position. We did not include input noise in this experiment, but rather introduced two new types of drift. In addition to *drift* (drift sections indicated by red bar and acts on spaceship), there was *invisible* drift (red bar was not shown, but drift acts on spaceship) and *fake* drift (red bar was shown, but drift did not act on the spaceship).

Experiment 3 was organized into three blocks, so that one new drift type was encountered at a time. We included a normal drift block as control condition that was always presented first. Within this block, half of the drift sections were normal drift and the other half had no drift. In the invisible block, only half of the drift sections in every trial were invisible, with the other being normal drift. In the fake block, again, only half the drift sections were fake and the other half were normal drift. The order in which the invisible and fake block were presented was counterbalanced across participants. Each layout was played at least twice within a block. Once with four random drift sections as no, invisible, or fake drift and the other four as normal drift. And the other time with the drift types reversed, so that the drift sections that were normal drift before were now no, invisible, or fake drift. This adds up to a total of 36 configurations. We included another training configuration with normal drift only in between blocks to establish a baseline.

During gameplay, participants saw only a small section of the entire environment drawn on the screen, the observation space (enlarged section on the left of Fig. 1). This is done to prevent longer‐term planning and promote situated action. It also enables associating eye movements with a given instance.

The following procedure was used across all three experiments.

### 2.3 Procedure

Once the height of the table had been adjusted and participants had found a comfortable position, the eye tracker was calibrated using 9‐point grid. Participants were instructed about the objective of the Dodge Asteroids environment and told that they will encounter difficulties (no explicit instruction of input noise or drift was given). Immediately afterward, participants entered training to familiarize themselves with controlling the spaceship. The random generated layout exclusively for training had a length of 18,000 pixels and featured the statistics of the easy difficulty. In Experiments 1 and 2, input noise of standard deviation 3.0 occurred halfway through the training layout. Likewise, drift was enabled from halfway through the training layout in Experiments 1 and 3. In all three experiments, participants had a total of three attempts to complete the training configuration without crashing. After completion of the training, the experiment started. A recalibration using a 5‐point grid was carried out prior to each trial. Participants started the recalibration themselves by pressing the space bar, which was always immediately followed by the start of the next configuration. At the start, the spaceship flew in from the top of the horizontal center screen until it reached screen x‐ and y‐coordinates 954 and 270 above the vertical center of the screen (coordinates refer to the top left corner of the spaceship sprite). From this point onward, participants were able to control the spaceship via key press. Key presses would not result in the spaceship moving across the screen, but the environment moving around the spaceship so that it remained static at all times. For reference, it took roughly 2.45 s for an object that appeared on the bottom to disappear again at the top of the screen. The static center position of the spaceship was chosen to be at eye level.

Regardless of completion or crash, a question assessing general control during steering was presented on the screen after each configuration (JoC; “In the most recent run, how strong was your feeling of control?”). Participants indicated their degree of control by pressing one of the number keys 1–7. A short break was possible after each trial allowing participants to disengage from the chin rest. The order in which the different configurations were played was fully randomized. If a configuration was completed, it was removed from the list of configurations to be played. Otherwise, if a crash occurred, the trial was abandoned and mixed back into the list of configurations to be played. For each configuration, participants were given only three attempts to prevent familiarization with specific instances within the layout. If all three attempts ended in a crash, the configuration was removed from the list of configurations to be played and not presented again.

On average, completing a layout of 9000 pixels length took 29.08 s (2.71), while a layout of 13,500 pixels length took 43.56 s (3.99), and a layout of 18,000 pixels length took 58.59 s (2.07). Deviations can occur because individual frames may last longer than $\frac{1}{60}$ s, depending on how many objects need to be drawn on the screen. In Experiment 1, the average total playing time across all participants was 1867.08 s (120.99). In Experiment 2, it was 2061.13 s (189.11). And, in Experiment 3, the average total playing time was 1549.71 s (92.68).

Once the list of configurations to be played was empty, the main part of the experiment ended, and participants were able to disengage from the chin rest for good. They were given a pen‐and‐paper questionnaire about video game expertise (Nolan, 2022) that was followed by a semi‐structured interview. The interview questions related to whether a total loss of control was experienced while steering the spaceship and what specific difficulties were encountered that may have led to a loss of control. Afterward, participants were debriefed.

The whole procedure was meant to never exceed a duration of 1.5 h.

### 2.4 Recruiting and exclusion criteria

Participants were recruited from the pool of students at the University of Potsdam using the SONA platform. Participants had normal or corrected to normal vision. Participation was compensated by receiving credit points. Each participant provided written informed consent. Not completing the training configuration within three attempts was an exclusion criterion that resulted in no actual exclusion.

Experiment 1 was conducted in the period from January 24 to March 31, 2023, in which a total of 27 participants were recruited. The six participants in Experiment 2 were recruited in the period from May 21 to June 07, 2024. For Experiment 3, we gathered the data of a total of 26 participants in the period from May 30 to June 15, 2023. The average age of participants across all three experiments was 23.57 years (SD = 4.89 years). No participant was allowed to participate in multiple of the experiments.

All three studies were conducted in accordance with the Declaration of Helsinki.^2^

### 2.5 Eye‐tracking

Eye movements were recorded binocularly using the ViewPixx TRACKPixx eye tracker (VPixx Technologies, Saint‐Bruno, QC, Canada) with a sampling rate of 2000 Hz. The eye tracker recorded *X*‐ and *Y*‐axes coordinates of the individual eyes of the participants. To obtain the final gaze position, we computed the Euclidean center point of both eyes. Binocular tracking allowed us to approximate the gaze position to the signal of the other available eye if the signal of one eye was lost.

While navigating the Dodge Asteroids environment (not during instructions or calibrations), a gray bar was displayed at the bottom of the screen, ranging the whole width of the screen with height 270 pixels. This prevented participants from looking outside the screen when positioning the gaze toward the bottom edge of the environment where new objects appeared.

Fixation detection within the data samples of the eye‐tracker was done for every experiment individually and by using a velocity‐based algorithm. Rows were annotated as fixation when both eyes traveled no more than 0.026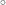 (1.25 pixels) between samples for a minimum of 25 consecutive samples (VPixx Technologies Inc., 2024). These may also have included smooth pursuit eye movements, since if the gaze was not aimed directly at the spaceship, the fixated point moved due to how we implemented movement on the screen visually. We verified the remaining data in a follow‐up step by applying a velocity‐based saccade detection algorithm (Engbert & Kliegl, 2003; Engbert & Mergenthaler, 2006). Here, rows were annotated as saccade when both eyes traveled at least 0.5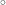 for a minimum of four consecutive samples (2.00*10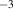 ms). We used a multiplier λ = 6 to compute the velocity threshold. This way, we detected a total of 96,263, 31,505, and 137,190 fixations for Experiment 1, 2, and 3, respectively.

**Fig. 2:**
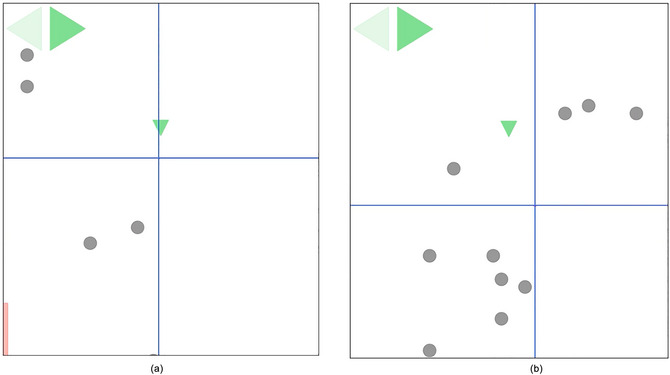
Simplified illustration of two separate instances of the Dodge Asteroids game environment in Experiment 1. The spaceship is indicated by the small green triangle. The blue cross indicates the fixation location. (a) shows a Close Fixation being executed within a 5.70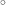 radius around the spaceship. (b) shows a Distant Fixation being executed outside a 5.70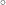 radius of the spaceship. In both cases, the participant is steering to the right indicated by the highlighted green arrow. Note that in (a), gaze is directed toward the immediate space in front of the spaceship, whereas in (b), gaze is located farther away in empty space, likely at a point along the planned trajectory of the spaceship within the environment.

We had to bear in mind that fixations that foveate the spaceship on the one hand or the surroundings on the other hand have different functions. If the spaceship is within high‐accuracy vision, the main focus lies probably on monitoring the individual movements of the spaceship in the immediate vicinity. On the contrary, if the gaze is shifted away from the spaceship and placed within the more distant environment, the main focus is most likely on visual exploration or the pursuit of a specific action goal, to which the gaze is primarily directed. We, therefore, hypothesized a distinction between two types of fixations based on the visual region in which the spaceship is located. *Close* Fixations keep the spaceship within the parafovea thus within a radius of roughly 5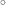 of visual angle from the exact point of fixation at the time it is initiated. Fixations that are initiated further away than roughly 5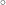 of visual angle from the spaceship (spaceship within peripheral vision) are labeled *Distant* Fixations (Fig. 2). We verified splitting the data using a cluster algorithm, the K‐quantiles clustering algorithm (Hennig, Viroli, & Anderlucci, 2019), which is a special case of k‐means. We applied K‐quantiles by using the QuClu package (Hennig, Viroli, & Anderlucci, 2022) in R (R Core Team, 2022). We set k, the number of clusters to be identified = 2, b, the number the initialization process is repeated = 50, and used the default, unconstrained method VS (Variable‐wise theta and Scaled variables). The only data dimension passed to the algorithm was the distance to the spaceship. For the data of Experiment 1, the algorithm identified a threshold value at 5.70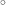. For Experiment 2, the threshold was identified at 5.65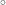. In the data of Experiment 3, the threshold was identified at 5.00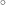. The respective data set was split into Close and Distant Fixations on the basis of the clusters found by advanced K‐quantiles.

Hypothesis testing was done individually for Close and Distant Fixations.

### 2.6 Measures

Completion is a variable with binary outcome that represents having completed the configuration. True in the case of managing to cross the finish line at the bottom of the environment and False in the case of a crash. For predicting completion, we included the features of the configuration as covariates (difficulty, input noise level, drift presence, drift type).
Judgment of control (JoC) is the term used for the responses to the general control question after each trial. Participants reported their judgments using a 7‐point Likert scale. JoC responses were predicted by the features of the configuration (difficulty, input noise level, drift presence, and drift type). We included two additional covariates: first, the number of fixations executed during the current trial; and second, the number of consecutive crash‐completions prior to the current trial, defined as the number of times a crash occurred followed by a completion of the very next trial.
Distance Measures. Distance to Spaceship & Distance to Closest Obstacle were derived from the fixation location at the point in time when the fixation was initiated. For both Close and Distant Fixations, the fixation location was specified regarding the distance to a reference point in degrees of visual angle. The reference point was either the center of the spaceship or the center of the closest obstacle. In models predicting the Distance to Spaceship or Distance to Closest Obstacle, we included input noise level or drift type covariates. We also included the number of obstacles and the number of drift sections visible on screen in the first frame the fixation was detected. With the last two variables, we accounted for the complexity of the visual scene.

### 2.7 Data analysis

We applied (generalized) linear mixed modeling using the MixedModels package (Bates, 2015) within the Julia programming language (Bezanson, Edelman, Karpinski, & Shah, 2017).

A Box–Cox distributional analysis (Box & Cox, 1964) with the MASS package (Venables & Ripley, 2002) in R indicated that several transformations are required. These were: a logarithmic transformation for distance to the closest obstacle in Distant Fixations in all three experiments; a square root transformation for distance to the spaceship in Close Fixations in Experiments 2 and 3; a reciprocal transformation for distance to the spaceship in Distant Fixations in Experiments 1 and 3; a reciprocal of the logarithmic transformed value for distance to the spaceship in Distant Fixations in Experiment 2. Lastly, due to the binary nature of the completion variable, we chose a Bernoulli link function when predicting the probability to complete a trial.

Maximal allowed random effects structures were explored for each model individually (Barr, Levy, Scheepers, & Tily, 2013). Including as many random effects as the experimental design justifies minimizes the risk of Type I errors by accounting for variance that might otherwise confound the variables of interest. This makes the effects found for variables of interest more robust against retesting. Model selection of the random effects structures was based on the Bayesian information criterion (Chakrabarti & Ghosh, 2011). Fixed effects structures were not subjected to a selection process and kept for hypothesis testing. All models were fitted using the maximum likelihood criterion. Hypothesis testing is based on z‐scores applying a |z|≥2.0 significance criterion. In addition to z‐scores, we report lower and upper bounds of the 95% highest density confidence interval. Confidence intervals were obtained using a parametric bootstrap with 10,000 replications based on the final selected model.^3^

### 2.8 Hypotheses

We hypothesize that in Experiment 1, increasing input noise will lead to fixations being initiated closer to the spaceship (H1.1). The reason for this is that input noise will elicit a growing number of prediction errors in visual sensory states and consequently not meeting the consistency criterion. This will prompt participants to select action goals with shorter time horizons that are also accounted for in gaze allocation. Additionally, in trials with higher input noise, the resulting rise in prediction errors and failure to meet the consistency criterion are expected to lead to control loss in participants, indicated by decreased JoCs (H1.2). Similarly, we expect that the probability to complete a layout (Completion Probability) is negatively impacted by increased input noise (H1.3).

Drift imposes unintended movement on the spaceship. However, given that drift sections are indicated by a red bar, we expect participants to anticipate these situations. Thus, they will allocate their gaze further away in front of the spaceship toward these situations (H1.4). We further hypothesize that there will be no significant difference in the JoC between trials with and without drift, as the anticipation of drift situations and the subsequent behavior, where participants can easily engage in proactive adaptation of action control, will help to prevent any control loss (H1.5). Furthermore, we do not expect any differences in Completion Probability between trials with and without drift (H1.6).

In Experiment 2, we expect that with increasing input noise, participants will initiate fixations at distances progressively closer to the spaceship (H2.1). Likewise, trials with increasing input noise will be associated with lower JoCs (H2.2) and decreased Completion Probability (H2.3).

In Experiment 3, we hypothesize that, compared to the normal block, the induced uncertainty in the invisible block will prompt participants to execute fixations at smaller distances to the spaceship (H3.1). This is based on adapted action selection and participants attending prediction errors occurring when drift suddenly shifts the spaceship. We further expect that trials within the invisible block are associated with lower JoCs, as prediction errors should occur in invisible drift situations, which could lead to the consistency criterion being challenged (H3.2). This will also lead to a decreased Completion Probability in trials within the invisible block compared to trials within the normal block (H3.3). The fake block as a control condition has visual indicators for drift sections, but these are not always reliable. We hypothesize that once this has been learned, there will be no loss of control, as it should be easy to adapt generated predictions which are then based solely on the own actions. Therefore, we expect that fixations during the fake block have distances to the spaceship similar to those in the normal block (H3.4: no significant difference in the distance to spaceship between fake and normal block). Likewise, we expect no differences in JoCs (H3.5) or Completion Probability (H3.6) between trials within the fake and normal block.

## 3 Results

To get a good balance in terms of JoCs and post‐experiment interviews, we aimed for a 50% chance of completing a configuration of layout and experimental manipulations in the piloting phase. Across all experiments, on average, participants completed a configuration in 58.9% (9.4%) of times with a minimum completion rate of 41.9% and a maximum completion rate of 77.3%. Available interview data about the experience of total control loss and the factors to which a control loss was attributed (keeping head static in chin rest, accurate description of input noise), as well as gaming experience, were explored as possible random intercept effects using null models of the various predicted variables. None of the factors explained an adequate amount of variance and we resorted to including participant ID as random intercept effect in every model we built.

### 3.1 Experiment 1

Due to technical errors, interview data of one of the participants in Experiment 1 was lost. In two out of 26 available interviews, participants hinted toward input noise as disturbance but were not able to accurately describe its effects. Given descriptions included time delay between key press and spaceships' horizontal movement and halting visualization. Two participants were able to accurately describe the effects of input noise (“varying steps,” “varying displacement in every game tick”). In the remaining 21 interviews, input noise or factors relating to input noise were not mentioned as causes for control loss. In two interviews, solely the number of obstacles was stated to reduce control. Solely the presence of drift was mentioned 11 times. The combination of both were stated eight times to have caused a loss in control. Out of the four times input noise was mentioned or hinted toward, two times all three factors (many obstacles, drift, and input noise) were mentioned with one time input noise being accurately described and one time it being described as delays. The other two times input noise was mentioned as the sole factor causing control loss, again once being accurately described and the other time it being described as halting visualization.

**Fig. 3:**
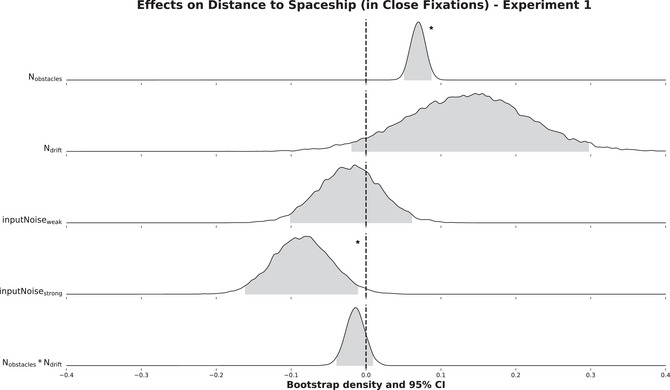
Distributions of β values for all covariates obtained from a parametric bootstrap with 10,000 iterations of the final selected model. The model predicted the distance between Close Fixations of Experiment 1 and the spaceship in visual degrees (no transformation required for the predicted variable). The gray shaded area in the distributions indicates the 95% ‐ highest density confidence interval. Significant effects are labeled with an asterisk.

Fig. 3 shows the distribution of β values obtained by a parametric bootstrap with 10,000 iterations when predicting the distance to the spaceship in Close Fixations. The number of obstacles on screen significantly increased the predicted variable (β = 0.07, σ = 0.01, 95% CI 0.05 to 0.09, z = 7.30). In contrast, in comparison to weak input noise, strong input noise significantly decreased the distance to the spaceship (β = −0.09, σ = 0.04, 95% CI −0.16 to −0.01, z = −2.19). Neither the number of drift sections (β = 1.14, σ = 0.08, 95% CI −0.03 to 0.28, z = 1.70), nor weak input noise (in comparison to no input noise; β = −0.02, σ = 0.04, 95% CI −0.10 to 0.06, z = −0.53) influenced the predicted variable.

When predicting the distance to the spaceship in Distant Fixations, we also found a significant increase in the predicted variable with increasing number of obstacles (β = 1.03*10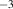, σ = 1.51*10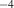, 95% CI 7.52*10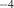 to 1.35*10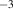, z = 6.85). There was no significant main effect for the number of drift sections (β = −3.60*10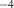, σ = 1.06*10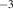, 95% CI −2.41*10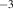 to 1.74*10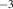, z = −0.34). Similarly, either level of input noise did not affect the distance to the spaceship in Distant Fixations (weak: β = 8.35*10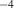, σ = 1.10*10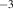, 95% CI −1.36*10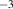 to 2.95*10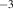, z = 0.76; strong: β = 3.72*10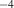, σ = 7.47*10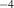, 95% CI −1.12*10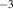 to 1.79*10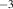, z = 0.50).

Having successfully completed the trial significantly increased JoCs (β = 1.52, σ = 0.16, 95% CI 1.19–1.82, z = 9.34). The following results stem from a model with Completion entered as random intercept effect. Increasing the difficulty of the layout to hard by increasing the total number of obstacles in the layout significantly decreased JoCs (β = −0.44, σ = 0.10, 95% CI −0.64 to −0.24, z = −4.30). Likewise, strong input noise significantly decreased JoCs (β = −0.34, σ = 0.06, 95% CI −0.46 to −0.21, z = −5.38). We found additional positive effects on JoC for the number of fixations executed throughout the trial (β = 1.16*10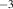, σ = 3.61*10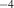, 95% CI 4.58*10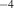 to 1.87*10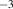, z = 3.22), and the number of times the participant crashed prior to the current trial but immediately after completed a layout (consecutive crash‐completion; β = 0.03, σ = 7.18*10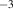, 95% CI 0.01–0.04, z = 3.64). Also, hard layout difficulty and the presence of drift interacted negatively with each other (β = −0.68, σ = 0.14, 95% CI −0.95 to −0.41, z = −4.92). Ultimately, the presence of drift in a given trial did not affect JoCs (β = −0.27, σ = 0.14, 95% CI −0.38 to −0.03, z = −1.87).

The probability to complete a layout was negatively affected by increased difficulty of the layout (medium: β = −2.17, σ = 0.64, z = −3.39; hard: β = −3.64, σ = 0.62, z = −5.90) and strong input noise (β = −0.84, σ = 0.19, z = −4.42). Note that the presence of drift did not influence Completion Probability (β = −0.28, σ = 0.79, z = −0.35).

### 3.2 Experiment 2

This experiment featured a total of five levels of input noise. In the subsequent interviews, all six of the participants were able to describe input noise accurately. Four participants mentioned that slight stuttering of the game (most likely due to input noise levels up to 6) was initially mistaken for technical problems with the screen. However, when more severe inaccurate controls (input noise of 9 and 12) began to occur periodically during the experiment, they realized that it was part of the experiment.

**Fig. 4:**
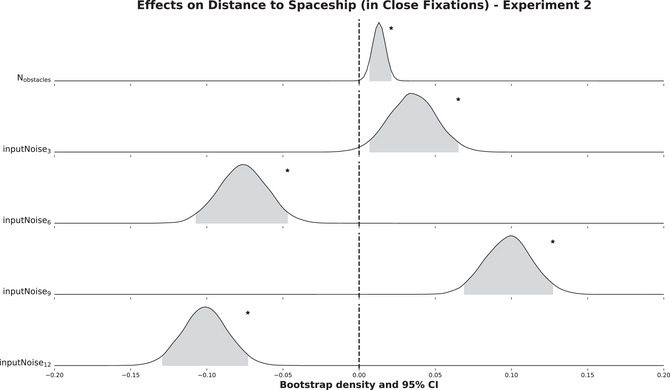
Distributions of β values for all covariates obtained from a parametric bootstrap with 10,000 iterations of the final selected model. Here, the model predicted the distance between fixation and spaceship in Close Fixations of Experiment 2. The predicted variable was square root‐transformed and is given in visual degrees. The gray shaded area in the distributions indicates the 95%‐highest density confidence interval. Significant effects are labeled with an asterisk. The subscripts of the input noise covariates refer to the standard deviation in pixels of the normally distributed disturbance when steering the spaceship. Thus, larger subscripts mean increased induced uncertainty in sensorimotor control. The contrasts were chosen so that each level of input noise is compared against the previous one.

Here, when predicting the distance to the spaceship in Close Fixations, we found that the number of obstacles on screen significantly increased the predicted variable (β = 0.01, σ = 3.07*10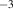, 95% CI 6.73*10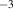 to 0.02, z = 4.25). For the input noise, we have chosen the contrasts so that each level is compared with the previous level. We found nuanced effects for every individual level; refer to Fig. 4 for a better overview. Compared to the intercept of no input noise, input noise:3 increased the distance to the spaceship (β = 0.04, σ = 0.02, 95% CI 3.99*10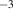 to 0.07, z = 2.24). But compared to input noise:3, input noise:6 decreased the distance to the spaceship (β = −0.08, σ = 0.02, 95% CI −0.11 to −0.05, z = −4.83). However, the comparison of input noise:9 against input noise:6 yielded an increase again (β = 0.10, σ = 0.02, 95% CI 0.07–0.13, z = 6.36). And finally, compared to input noise:9, input noise:12 significantly decreased the distance to the spaceship again (β = −0.10, σ = 0.02, 95% CI −0.13 to −0.07, z = −6.90).

Similar to the effect in Close Fixations, the number of obstacles increased the distance to the spaceship in Distant Fixations (β = 1.02*10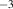, σ = 1.52*10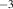, 95% CI 7.26*10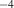 to 1.33*10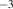, z = 6.72). Compared to no input noise, input noise:3 significantly decreased the predicted variable (β = −2.45*10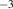, σ = 1.03*10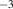, 95% CI −4.48*10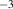 to −4.70*10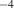, z = −2.38). The distance to the spaceship further decreased in response to input noise:6 (β = −3.75*10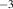, σ = 1.09*10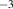, 95% CI −5.90*10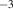 to −1.64*10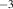, z = −3.45). Compared to input noise:6, input noise:9 then led to an increase in the predicted variable (β = 3.64*10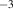, σ = 1.07*10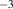, 95% CI 3.64*10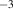 to 1.07*10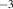, z = 3.41). Further increasing input noise to 12 yielded no significant difference to input noise:9 (β = 1.01*10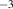, σ = 1.05*10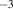, 95% CI −1.05*10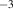 to 1.05*10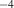, z = 0.97).

Successfully completing a trial in Experiment 2 led to an increase in JoCs (β = 1.74, σ = 0.30, 95% CI 1.14–2.31, z = 5.84). Of the input noise levels, only input noise:9 led to a significant decrease in JoCs compared to input noise:6 (β = −0.89, σ = 0.22, 95% CI −1.33 to −0.45, z = −3.98). Although there is a negative trend across all input noise levels, no other comparison yielded significance. Interestingly, we also found no other effects for covariates that we explored in Experiment 1, such as increased difficulty of the layout (β = 0.13, σ = 0.14, 95% CI −0.15 to 0.41, z = 0.93), the total number of fixations layout (β = 5.69*10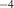, σ = 8.59*10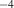, 95% CI −1.21*10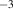 to 2.23*10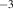, z = 0.66), or the number consecutive crash‐completions (β = 3.55*10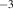, σ = 0.02, 95% CI −0.05 to 0.05, z = 0.15).

Completion Probability in Experiment 2 decreased when layout difficulty increased (β = −1.13, σ = 0.32, z = −3.49). Imposing weak (3 pixels SD) or (6 pixels SD) moderate input noise had no effect on Completion Probability (input noise:3, β = −0.08, σ = 0.67, z = −0.12; input noise:6, β = −0.11, σ = 0.64, z = −0.17). But in comparison to input noise:6, input noise:9 significantly decreased Completion Probability (β = −1.46, σ = 0.53, z = −2.73), with input noise:12 further decreasing Completion Probability (β = −1.05, σ = 0.38, z = −2.75).

### 3.3 Experiment 3

Experiment 3 featured new types of drift sections, namely, invisible and fake drift, which participants encountered in corresponding experimental blocks.

In the semi‐structured interviews following the experiment, only four of the 26 participants noticed and described fake drift. And only three of these confirmed that they felt their control over the spacecraft was restricted by fake drift. All but one participant mentioned invisible drift as a factor limiting their control.

When predicting the distance to the spaceship in Close Fixations, the distance increased with every additional obstacle on screen (β = 0.01, σ = 2.77*10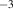, 95% CI 0.01−0.02, z = 4.48). No other significant main effects were found. Neither the number of drift sections on screen (β = 1.11*10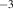, σ = 7.43*10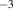, 95% CI −0.01 to 0.02, z = 0.15), nor the comparison of any of the two new drift types versus normal drift yielded significance (invisible block: β = −0.03, σ = 0.02, 95% CI −0.07 to 0.02, z = −1.17; fake block: β = −0.02, σ = 0.03, 95% CI −0.07 to 0.03, z = −0.82).

The model predicting the distance to the spaceship in Distant Fixations revealed a similar effect for the number of obstacles on screen as in Close Fixations. Here again, an increasing number of obstacles is associated with an increasing distance to the spaceship (β = 7.99*10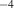, σ = 1.03*10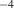, 95% CI 6.02*10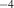 to 1.01*10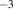, z = 7.75). However, in contrast to Close Fixations, the number of drift sections on screen significantly increased the distance to the spaceship in Distant Fixations (β = 4.45*10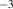, σ = 7.65*10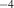, 95% CI 2.94*10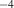 to 5.93*10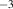, z = 5.82). Yet, neither type of drift showed a significant effect on the predicted variable (invisible block: β = 3.40*10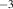, σ = 2.58*10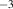, 95% CI −2.77*10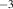 to 1.87*10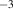, z = 1.32; fake block: β = 1.22*10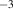, σ = 4.06*10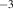, 95% CI −7.04*10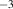 to 8.95*10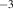, z = 0.30).

**Fig. 5:**
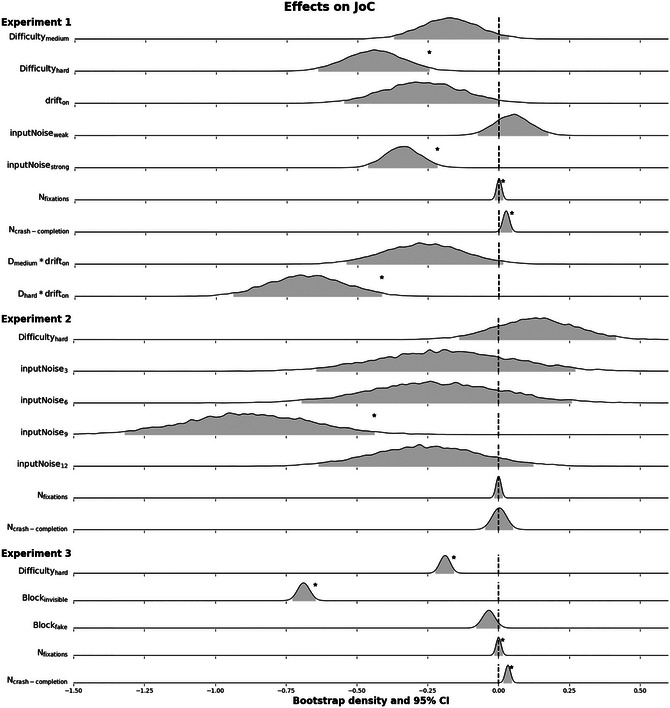
Distributions of β values for all covariates obtained from a parametric bootstrap with 10,000 iterations. The parametric bootstraps were based on the final selected model in the respective experiment. Each of the models predicted the control judgment responses (JoC; possible responses ranged from 1 to 7) given by participants after every trial. No transformations were required, which simplifies the comparability of the effects across experiments. The gray shaded area in the distributions indicates the 95%‐highest density confidence interval. Significant effects are labeled with an asterisk. Note that $N_{\text{fixations}}$ positively influenced JoCs in Experiments 1 and 2, but the kernel bandwidth used for the kernel density estimates in generating the distributions was likely too wide to accurately capture its small effect.

Successful completion of trials also increased JoCs reported in Experiment 3 (β = 1.47, σ = 0.02, 95% CI 1.44–1.51, z = 81.65). Entering successful completion as random intercept effect into the model, we found that raising layout difficulty from medium to hard decreased JoCs (β = −0.19, σ = 0.02, 95% CI −0.22 to −0.16, z = −11.77). Further, JoCs in the invisible block were significantly lower than those in the normal block (β = −0.69, σ = 0.02, 95% CI −0.73 to −0.65, z = −34.54). There was no difference between JoCs in the fake and the normal block (β = −0.03, σ = 0.02, 95% CI −0.08 to 6.90*10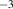, z = −1.66). Also here, in Experiment 3, we found positive effects for the number of fixations executed throughout a trial (β = 6.57*10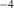, σ = 7.61*10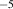, 95% CI 5.10*10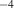 to 8.12*10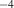, z = 8.63) and the number of consecutive crash‐completions (β = 0.03, σ = 1.97*10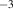, 95% CI 0.03−0.04, z = 17.11). For a better overview and comparison of the effects on JoC in all three experiments, refer to Fig. 5.

Surprisingly, Completion Probability was not affected by increased layout difficulty (β = 0.14, σ = 0.14, z = 0.98). However, compared to the normal block, in the invisible block, Completion Probability was significantly decreased (β = −0.91, σ = 0.17, z = −5.46). Lastly, Completion Probability was not affected by the fake block compared to the normal block (β = 0.12, σ = 0.18, z = 0.63).

### 3.4 Additional analyses

In further exploratory analyses, we tested what affects the distance to the closest obstacle in Distant Fixations across experiments. If we assume that the locations of Distant Fixations reflect action goals that are pursued (Heinrich et al., 2024), it can be informative to explore how action selection is adapted in order to be more efficient under control loss—for instance, by maintaining greater distances from obstacles when there is input noise or drift.

In Experiment 1, the predicted distance increased the more drift sections were on screen (β = 0.04, σ = 0.02, 95% CI 0.01–0.07, z = 2.59). Neither weak input noise (β = −3.69*10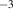, σ = 5.42*10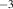, 95% CI −0.02 to 6.25*10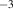, z = −0.68) nor strong input noise (β = 7.63*10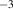, σ = 5.60*10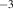, 95% CI −2.83*10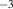 to 0.02, z = 1.36) had an effect on the distance to the closest obstacle.

In Experiment 2, input noise:3 led to an increase in the distance to the closest obstacle in Distant Fixations (β = 0.04, σ = 0.01, 95% CI 0.01–0.07, z = 2.88). Further, increasing input noise yielded no significant difference in comparison.

Contrary to the findings in Experiment 1, in Experiment 3, we found that the number of drift sections on screen decreased the distance to the closest obstacle (β = −0.11, σ = 0.03, 95% CI −0.17 to −0.06, z = −4.31). In turn, compared to the normal block, fixations in the invisible and fake blocks featured longer distances to the closest obstacle (invisible block: β = 0.03, σ = 0.01, 95% CI 6.51*10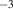 to 0.05, z = 2.50; fake block: β = 0.04, σ = 0.01, 95% CI 8.31*10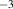 to 0.06, z = 2.68).

We conducted an additional analysis to examine how the proportion of Distant Fixations (and conversely, Close Fixations) within the total number of fixations changes as a function of the experimental manipulations.

In Experiment 1, we found no significant main effects. However, there was a significant interaction between high level difficulty and drift (β = −0.08, σ = 0.03, 95% CI −0.14 to −0.02, z = −2.51). When level difficulty increased from medium to high and drift was enabled, the proportion of Distant Fixations decreased, meaning that the proportion of Close Fixations increased.

This interaction could not be replicated in Experiment 3, and we found no effects for other types of drift. Likewise, in line with the results of Experiment 1, we observed no effects of input noise on the proportion of Distant Fixations in Experiment 2.

## 4 Discussion

In this series of studies, we investigated how motor perturbations of varying predictability—input noise and drift—impact performance, control judgments (JoC), and eye‐movement behavior in a dynamic, interactive environment. We differentiated between reactive and proactive strategies of action control by examining changes in participants' behavior while steering a spaceship. But it was only when we distinguished between different types of fixations that the results contributed to our understanding of the dynamics of situated action control and their link to shifts in the SoC.

Our analysis of fixation distances to the spaceship in Experiment 1 revealed mixed support for H1.1. As assumed, Close Fixations showed that higher levels of input noise reduced the distance to the spaceship, reflecting cautious behavior in response to control loss. However, in Distant Fixations, input noise did not affect fixation distances.

In Experiment 2, input noise also did not lead to a monotonic decrease in fixations distance to the spaceship (H2.1). In Close Fixations instead, we observed an effect, with each level of input noise oscillating in the opposite direction from the level before. We cannot exclude the possibility that these results are partly due to subjects being aware of input noise as an experimental condition. Our initial understanding of these results is that participants were actively trying to visually ignore the stuttering of obstacles in the environment and focus on the spaceship. However, the interpretation of these effects remains mostly unclear. In Distant Fixations, we found a successive reduction of the distance through the first two levels of input noise. Then, however, we found a slight increase in distance for input noise:9. Note that this increase did not go beyond the distance of the intercept of no input noise. These results better fit a step‐function than the assumed monotonic decreasing one: once uncertainty reached a critical threshold, participants adapted by shifting gaze closer to the spaceship, a reactive adaptation of action control, but further increases in noise did not exacerbate this effect. This threshold‐like response highlights the limits of sensorimotor compensation under unpredictable conditions.

The increased distance to obstacles observed under input noise in Distant Fixations of Experiment 2 further supports the idea of reactive adaptation of action control. By prioritizing immediate safety and feasibility, participants demonstrated a shift in action goals under uncertainty, pursuing those more likely to succeed. This suggests that participants engaged in reactive adaptation of action control to cope with unpredictable disturbances, prioritizing immediate control over projecting their environment into the more distant future. The action selection process is adapted in terms of its top‐down information processing component. Initially, agents were conservative in their top‐down control, relying primarily on sensorimotor processing with action selection largely driven bottom‐up. However, during control loss, top‐down control—and consequently cognitive processing—increases. Planning becomes more incremental to maintain high performance.

The conclusion that reactive adaptation of action control is based on an awareness boundary, as Kahl et al. (2022) assume, is further supported by the fact that JoC did not decrease linearly with increasing input noise but instead followed a threshold function. Once participants experienced control loss, additional input noise did not further reduce their SoC (contrary to H1.2 and H2.2). These findings suggest that control judgments are influenced more by cognitive processes, such as explaining the environment, than by sensorimotor compensations for disturbances.

We could not confirm that gaze is allocated further away in front of the spaceship in drift situations in Experiment 1 (H1.4). Yet, particularly changes in the statistics of Distant Fixations in Experiment 3 indicated that participants learned and adapted their behavior in response to predictable disturbances, even in the absence of visible feedback. Here, the number of drift sections on the screen increased the distance to the spaceship in Distant Fixations, supporting H1.4, which we stated for Experiment 1. But we do have to reject H3.1, as the invisible block had no effect on distances between fixation and spaceship. We would have expected participants to allocate their gaze closer to the spaceship, as invisible drift can occur suddenly and unexpectedly and that would create immediate demands for maintaining the spaceships trajectory similar to high input noise conditions. Further exploratory analyses of Distant Fixations revealed that number of drift sections decreased distance to the closest obstacle in Experiment 3. Only within the invisible drift block, fixations featured increased distances to closest obstacles. This suggests that the presence of a mental model of environmental features like drift enables proactive strategies, allowing participants to anticipate and prepare for sudden perturbations. The results for drift in general provided support for proactive adaptation of action control. Note that fake drift had no effect on the distance to the spaceship (H3.4) or to the closest obstacle, suggesting that proactive adaptation of action control is used to mitigate conditions that are detrimental and arise when the agent does not have an accurate model of the environment.

As hypothesized, normal drift did not affect JoC (H1.5). This lack of impact suggests that proactive adaptation of action control prevented participants from experiencing a loss of control. Fake drift probably had no effect on JoC (H3.5), as this type of drift did not result in a loss of control over the spaceship. Only when drift became unpredictable and actually affected the spaceship, as in the invisible drift condition, did participants report control loss, aligning with H3.2. Taken together, this indicates that control loss arises not from the drift disturbance itself but from the inability to link the perceived disturbance to the mental model of the environment.

We explored other variables that could be integrated when judging the own SoC. We found that in Experiments 1 and 3, participants reported higher JoCs for every additional fixation they executed or for every time they successfully completed a trial after crashing (Fig. 5). However, this effect was observed only in experiments, which featured predictable drift and is missing in Experiment 2, which only featured unpredictable input noise. This may be due to the fact that the SoC can be improved by adapting one's behavior (oculomotor or steering) in response to predictable changes in the environment. This again supports that proactive adaptation of action control is based on anticipations and projections of the environment.

The inability to explain perceived disturbances based on a mental model of the environment was linked to poorer performance. For instance, random noise in control over the spaceship and invisible drift led to reduced Completion Probabilities, as predicted by H1.3, H2.3, and H3.3. This highlights the importance of predictability in supporting effective action control and maintaining performance. However, this effect is most likely because the situations that were caused by inaccurate models of the environment were detrimental, supported by the fact that fake drift did not affect performance (H3.6).

An additional analysis provided further insight into the relative use of Close versus Distant Fixations. Both types of fixational eye movements are crucial for action control, which may explain why we did not observe consistent differences in the proportion of Distant Fixations across experimental manipulations. This indicates that participants flexibly balance both fixation types depending on task demands, maintaining a robust allocation strategy even under varying levels of noise and drift. The significant interaction between hard level difficulty and drift in Experiment 1, however, shows that this balance can shift under particularly demanding conditions, most likely within drift sections where immediate control needs rise sharply, especially when many obstacles are present. The absence of this effect in Experiment 3 points to context‐dependence and highlights boundary conditions (obstacle density) that may be worth investigating to determine under which circumstances changes in fixation proportions emerge.

This work highlights visual focus as an important measure for shifts between reactive and proactive strategies in situated action control, when people respond to environmental features of varying predictability. However, the role of fixation distances to a reference point in action goal pursuit under (un)certainty needs further study. Gaze metrics should be assessed not just as static measures. Examining how gaze transitions between regions could offer deeper insights into the link between the SoC and cognitive processes. Close Fixations seem to represent immediate control needs, while Distant Fixations may signal readiness for visual exploration and proactive adaptation of action control. This distinction aligns with the observed effects of experimental manipulations on these two types of fixations. Identifying especially the transitions between Close and Distant Fixations might be a key indicator to better understand action control holistically.

Our study has limitations in separating sensorimotor control from cognitive control processes. However, we believe that accounting for both levels and their interplay is essential to fully understand real‐world behavior. To achieve this, it is important to allow flexibility not only in how movements are controlled but also in how action goals are selected. Future research could explore the connection between these two levels by combining eye‐tracking with precise joystick tracking. For tasks involving reaching, devices that track hand or mouse movements can also provide valuable insights.

Additionally, future research should incorporate computational models to rigorously formalize how fixation distances relate to action control. We have already taken the first step in the direction of simulation‐capable models by using a type of data analysis that provides mathematical models. Such models are continuously adapted and can simulate various fixation locations and strategies over time. These simulations display how visuomotor behavior evolves, adapting to predictable and unpredictable features of the environment but being dependent on prior behavior. We believe that approaching action control from the perspective of dynamical systems is crucial for a better understanding.

In this last part, we would like to take the discussion beyond the scope of the experiments described here. We have not yet addressed the concept of multisensory perception. Prediction errors can occur in a variety of modalities, such as an action not producing the expected sound. Where it is easy to infer how prediction errors in visual perception cause adjustments in action goals (here measured by eye movements), it is much harder to discuss how auditory prediction errors (or even haptic or olfactory ones) influence action plans. According to multilayered models of action control such as that of Kahl et al. (2022), prediction errors detected in the sensorimotor system are integrated into a holistic representation of the action at the cognitive level (which includes the SoC). However, it is quite possible that the different modalities are not represented in equal proportions. Additionally, prediction errors in the different sensors may lead to different adaptations in action control and not to a certain adaptation to different degrees. The concept of the SoC refers to the overall control experience. This means that all prediction errors, regardless of the sensory origin, are integrated into a low‐level SoC (whether individual or same), which then translates into a single higher‐level SoC. This raises the question of what effects a haptic prediction error can be expected to have on the SoC compared to an auditory prediction error, for example. Because haptic feedback is more integral to precise motor actions (e.g., holding, pressing, balancing), we think that in comparison to auditory prediction errors, haptic ones will lead to a greater loss in control. There are interesting interactions to be explored as well. Like what happens to the SoC and action control when prediction errors occur in all modalities except visual perception, the modality we rely on the most? Also, how does the SoC play into the rich representation of the action and how specifically does it elicit adaptations in action control? Regarding time perception, if expected changes in the environment are brought about, but at the wrong time, this will certainly lead to adaptations on the temporal dimension of subsequent actions and anticipated action feedback. Finally, it may even be that the sensory origin of prediction errors primarily drives whether reactive or proactive adaptation of action control is applied.

Under the assumption that the current action goal influences where visual attention is allocated, we used eye‐tracking to implicitly measure action goals. As mentioned above (Section 1.3), eye‐movement behavior—the allocation of visual attention—is driven by multiple factors, which can broadly be categorized as either top‐down or bottom‐up processing. Action goals fall under top‐down (goal‐directed) processing, while strictly visual features such as edges, colors, and movement are assigned to bottom‐up (stimulus‐based) processing. Behavior at any moment results from a combination of both types, but they do not contribute equally. We propose that the SoC, reflecting how effectively one's past action goals have been implemented, determines how top‐down and bottom‐up processing are weighted in shaping behavior. When SoC is high, top‐down processing predominates because the pursued goal is well‐defined, supported by an accurate cognitive representation of the environment. In contrast, when SoC is low, bottom‐up processing has a stronger influence, meaning behavior is guided mainly by low‐level perceptual cues. The action goal is then only vaguely represented, lacking the precision needed to effectively drive behavior. Primarily, bottom‐up‐driven attention may support exploration of the surroundings while still solving the task at hand, helping to gather new evidence and refine the mental model of the environment.

Lastly, a potential long‐term goal might be to integrate SoC into autonomous systems. In this case, action selection would respond to real‐time changes in environmental conditions but also factor in previous action selection and the feasibility during execution. This approach can improve the fluency and efficiency of action control, and increase the system's overall capability and performance.

Overallvv, the findings underscore the adaptability of situated action control, illustrating how humans adjust their strategies to navigate complex and continuous environments. What we found contributes to our understanding of the general interplay between vision, motor control, and cognitive processes. Beyond these insights, we welcome discussions on the broader implications of our research, particularly how it relates to other domains of cognition and behavior.

## Funding

This research was funded by the German Research Foundation (DFG) Priority Program 2134 “The Active Self.”

## Notes

1
Git repository of the Dodge Asteroids experimental environment.

2 This research was reviewed and approved by the Ethics Committee of the Department of Psychology and Ergonomics (IPA) of the Technische Universität Berlin.

3 Data and analysis code, including model selection, is available in the following Git repositories: Experiment 1, Experiment 2, and Experiment 3.
